# Application of Ag/AgCl Sensor for Chloride Monitoring of Mortar under Dry-Wet Cycles

**DOI:** 10.3390/s20051394

**Published:** 2020-03-04

**Authors:** Yupeng Tian, Peng Zhang, Kaiyue Zhao, Zhenxing Du, Tiejun Zhao

**Affiliations:** 1 Center for Durability & Sustainability Studies, Qingdao University of Technology, Qingdao 266033, China; qdlgtyp@163.com (Y.T.); applezky@126.com (K.Z.); ztjgp@qut.edu.cn (T.Z.); 2 Department of Civil Engineering, Qingdao University of Technology, Qingdao 266033, China; 3 School of Materials Science and Engineering, Southeast University, Nanjing 211189, China; 230189193@seu.edu.cn

**Keywords:** reinforced concrete, corrosion monitoring, Ag/AgCl electrode, anode ladder monitoring system, dry-wet cycle

## Abstract

An Ag/AgCl electrode used as a corrosion sensor in a reinforced concrete structure is considered as having good application prospect. However, its performance under complex conditions, such as dry-wet cycle condition, is not affirmed. In the current study, the performance of Ag/AgCl as chloride selective electrode in mortar exposed to dry-wet cycle condition was investigated. A simple Ag/AgCl electrode was prepared and fabricated by electrochemical anodization. These Ag/AgCl electrodes were embedded into a mortar specimen with temperature sensors, humidity sensors and anode ladder monitoring system (ALS). After 28 d curing time, the upper surface of mortar specimen was wetted (with 5% NaCl solution) and dried regularly. The obtained results indicate that Ag/AgCl electrode responds to the ingress of chloride ion, sensitively. The chloride ion concentration variation can be reflected by the potential trend. Furthermore, the balance potential of Ag/AgCl electrodes is influenced by dry-wet cycles. Compared with ALS, it demonstrates that Ag/AgCl electrodes are more sensitive to chloride. The research provides the key element for the specific application of Ag/AgCl electrode for corrosion monitoring in the future.

## 1. Introduction

Reinforced concrete (RC) has been widely used in coastal environments, such as bridges, docks and offshore platforms, while its unexpected durability always baffles the construction industry [1,2,3]. Chloride-induced steel corrosion has commonly been accepted as the major reason for deterioration of RC structures [4,5,6]. In order to continuously monitor steel corrosion, adequate technology with high reliability to reflect chloride ion concentration is a vital element. For determination of distribution and variation of chloride ion in cement-based materials, there are mainly destructive and non-destructive testing methods [6,7]. With regard to non-destructive testing methods, in situ monitoring has the distinct advantage of real-time data acquisition [8,9,10]. Embedding Ag/AgCl electrode into cement-based material as chloride selective electrode (ISE) has a good application prospect. Ag/AgCl electrode is highly sensitive to chloride ions [11,12]. The potential response of Ag/AgCl electrode is stable in solution with a given chloride concentration. It is reported that the reference electrode prepared by electrolysis had a potential fluctuation of less than 2.7 mV in chloride solution with concentration of 0.1 mol/L [13]. In addition, Ag/AgCl electrode has the advantages of small temperature coefficient and good reversibility. In general, the common Ag/AgCl chloride electrode is a solid-state membrane electrode that consists of an Ag core with a coating layer of AgCl [14,15,16,17,18]. For the formation of outer AgCl layer, there are several commonly manufactured methods such as powder pressing, sintering [11,19], chlorination and DC current polarization [20].

With regard to application of Ag/AgCl, it has been used as the reference electrode for many purposes [15,20,21] and in recent decades has began to be studied in its use as chloride sensor in the concrete industry [17,22,23]. Relative attempts to embed Ag/AgCl electrodes into cement-based materials were gradually reported since the 1990s [24,25,26,27]. After that, many researches have been carried out. Guillem et al. [28] found that chloride ion activity coefficients in concrete inner-pore solutions may be calculated theoretically using Pitzer’s model based on the potentiometrically using ISE. Ueli Angst et al. [29] found that Ag/AgCl electrodes can be successfully used in highly alkaline environments, and it is only in the complete absence of chloride that the potential is affected by the pH. It is reported the relationship between response potential and the logarithmic functions of ionic activity range from 10 ^−4^ to 2 exhibits well linear [30]. Pargar et al. [31] systematically analyzed the reasons for the difference in working performance of different electrodes. Montemor et al. [32] prepared and studied a multiprobe chloride sensor in solution and also in mortar and concrete samples. Jin et al. [33] revealed that real-time monitoring of free Cl^−^ concentration in concrete was achieved based on the measurements of potentials of embedded electrodes and the diffusion law of free Cl^−^ content followed Fick’s second law.

Despite the results in these thoroughly elaborated works, the properties and performance of Ag/AgCl electrodes in the alkaline medium are yet to be affirmed, especially in view of their application as chloride sensors for RC structures. For RC structures, chloride often penetrates into their interior from the outside environment, which is a chloride uptake process. When concrete is exposed to the hydrostatic pressure, water penetrates into concrete by capillary suction. Although the water front and chloride penetration front are different due to the filtration effect [34], the penetration depth of water determines the penetration depth of chloride ions. As is known, most outdoor RC is inevitably subjected to dry-wet cycle condition [35,36]. The improvement of chloride ions permeation caused by dry-wet cycle leads to concrete facing higher risk of chloride-induced deterioration [37]. Accordingly, it is of importance to study the performance of Ag/AgCl electrode under dry-wet cycle condition.

The objective of the present investigation is to evaluate the performance of Ag/AgCl, as chloride ion sensor, for in situ monitoring of cement-based material under dry-wet cycle condition. As an extension of previous research [38], in this paper, Ag/AgCl electrodes as working electrode and MnO_2_ as reference electrode were first prepared. Reliability of Ag/AgCl electrode was tested by calibration. Ag/AgCl electrode system, anode ladder monitoring system (ALS), temperature sensor and humidity sensor were embedded in mortar sample. Dry-wet cyclic test was implemented by regular surface impregnation to accelerate the ingress of chloride into a sample. The performance of Ag/AgCl with chloride ingress will be present and discussed. By the results presented in this investigation, the efficiency of Ag/AgCl electrode application in practical cement-based material is evaluated.

## 2. Experimental Materials, Methods and Technical Background

### 2.1. Materials and Sample

Mortar cube specimen with dimensions of 170 (height) mm × 600 (width) mm × 900 mm (length) was manufactured using a water to cement ratio of 0.55. The amounts of cement, sand and water used per cubic meter were 515, 1350 and 283 kg, respectively. The cement used was Ordinary Portland Cement with 42.5 degrees. The natural river sand used was from Qingdao area, with a maximum diameter of less than 5 mm. No superplasticizer admixtures were added during the casting process. After demoulding, the tested specimen was placed in stable humidity and temperature condition at around 20 ± 3 °C and 60% RH. For the first 14 days of a total 28 days curing time, the specimen was cured by continuous pouring water. Then it was dried for 14 days to promote ingress of Cl^−^ into the specimen.

### 2.2. Preparation and Installation of Sensors

#### 2.2.1. Ag/AgCl Electrode

It is reported that a current density above 2 mA/cm^2^ may lead to AgCl layer heterogeneity and ionic resistivity [11]. In this paper, Ag/AgCl probes were fabricated by applying 0.5 mA/cm^2^ direct current (DC) on sterling silver (9.9 purity) with 2.5 mm diameters and 20 mm long for 2.5 h polarizations. Before DC polarization, raw Ag bar was pretreated as follows: 20 mm long Ag bars were zoned as 5 and 15 mm. The 5 mm end was used to connect copper conductor by welding. The welding zone was sealed with epoxy to prevent copper wire and Ag bar from electric couple corrosion. The 15 mm end to be polarized was polished with sandpaper (NO 1000) and was rinsed with acetone to remove residue and grease. Surface of Ag bar is vulnerable to be oxidized during polished process. In order to move the outer oxide layer, Ag bar was therefore immersed in 5% nitric acid solution for 60 s; then, Ag bar was orderly rinsed by ethyl alcohol and deionized water.

**Figure 1:**
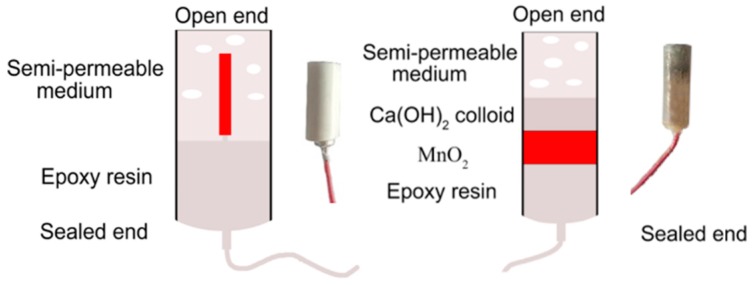
Package schematic diagram of Ag/AgCl and MnO_2_ electrode.

Considering the outer AgCl layer is vulnerable to being destroyed, a PVC tube with inner diameter of 20 mm was used to pack the electrode. Front end of prepared electrode 10 mm long was embedded in a semi-permeable film. This semi-permeable film, longwise, 25 mm long, is virtually composed of cement-based fillers (cement: sand: water: sawdust = 48: 48: 19.2: 2.5), which is able to protect the electrode and allow the pore solution to penetrate. The remaining 10 mm long end of prepared electrode was sealed by epoxy resin to fix the Ag/AgCl electrode. The schematic diagram of Ag/AgCl electrode is presented in Figure 1.

#### 2.2.2. MnO_2_ Electrode

MnO_2_ electrode has been commonly used as a reference electrode because of its good stability [39]. The composition of MnO_2_ electrodes used in this study includes manganese powder, manganese dioxide powder, carbon powder and polytetrafluoroethylene, with a ratio of 1:6:2:1. After the raw materials were mixed, the mixture was stirred with high-speed dispersing homogenizer and grinded into powder. MnO_2_ electrode (h = 5 mm, d = 10 mm) was prepared by direct compression method with 96 Mpa. PVC tube with an inner diameter of 10 mm was used as the shell of the electrode. Internal fills were subdivided into 4 layers, namely, mortar semi-permeable membrane (10 mm), alkalinegel (5 mm), MnO_2_ electrode (5 mm), epoxy resin (20 mm). The schematic diagram of MnO_2_ electrode is given in Figure 1. The temperature coefficient is 0.25 mV/°C, which shows that the temperature has little effect on the potential of the reference electrode. Some other details can be found in the previous study [38].

#### 2.2.3. ALS and Humidity Sensor

**Figure 2:**
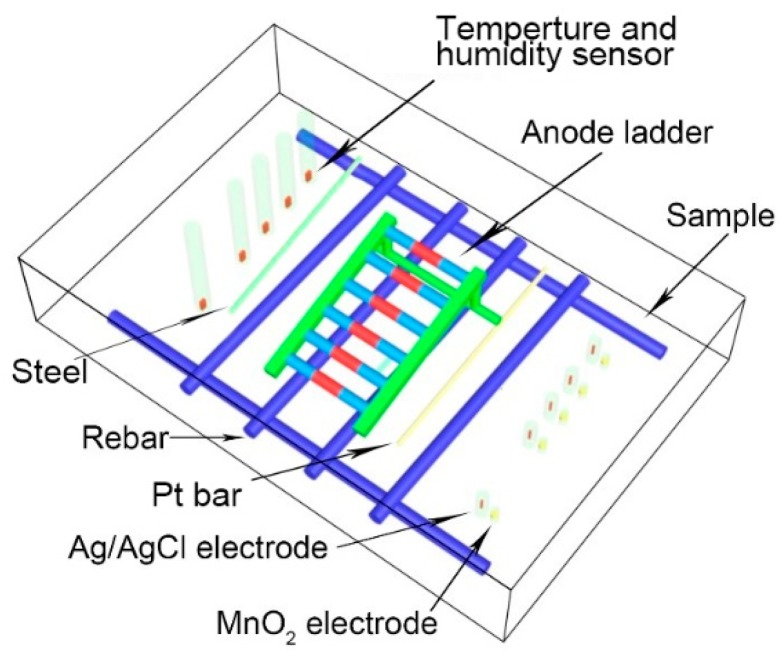
Position of Ag/AgCl electrode, MnO_2_ electrode, anode Ladder monitoring system (ALS), temperature sensor and humidity sensor in mortar sample.

The common ALS consists of six anodes and one cathode as showed in Figure 2. The so-called anodes are comprised by six common carbon steel bars, and the cathode is made of platinum-plated titanium rod. The ALS in this paper is supplied by Shanghai Lrel Instrument Equipment Co. in China. When the ammeter connects the anode carbon steel bars to platinum bar, the corrosion current can be orderly obtained. The corrosion potential of each anode was measured with a voltmeter by connecting the anode carbon steel bars to the platinum bar. Humidity sensors, which are the chip sensor from Qingdao Jiaqi Electron Equipment Co. in China, with an accuracy of 2%, helped explain the water penetration process.

#### 2.2.4. Installation and Position of Sensors

If the chemical composition of the electrolyte near ISE is different from that around the reference electrode, the diffusion potential will become an error source [27]. To avoid error from diffusion current, which is possibly induced by location difference with pH between the working electrode and the reference electrode, the MnO_2_ electrode was positioned as close Ag/AgCl probe as possible. In order to simulate the application of the ALS in the real environment, the ALS was mounted on a steel mesh, which was made by HPB300 carbon steel with a diameter of 12 mm. Specific installation location is shown in Figure 2. With regard to installation of humidity sensor, PVC tubes with 15 mm diameters were fixed to ensure the sensors at the designated location in the specimen. Furthermore, such positioning measures avoid humidity sensor to contact with water, which may result in failure of the chip sensor. After 28 days curing time, the humidity sensor was sealed into the reserved PVC tubes. The detailed location information of sensors from the sample surface is given in Table 1.

### 2.3. Calibration in Solution

The synthetic pore solution was obtained by diluting NaCl into saturated Ca(OH)_2_ solution. Five types of synthetic pore solution with 1, 0.5, 0.1, 0.01, 0.001 M NaCl were used to calibrate the potential of Ag/AgCl sensor. The electrode potentials were tested at a constant temperature of 20 ± 1 °C. The tested electrode was rinsed after each measurement to avoid residual solution influence response time.

### 2.4. Application of Ag/AgCl Electrode in Mortar

In order to expedite ingress of chloride into sample, dry-wet cyclic condition was conducted by 5% (by mass) NaCl solution. During the wetting phase, NaCl solution was continually poured on the upper surface of the sample to maintain a liquid level 2 mm high than sample surface height. Correspondingly, drying process was accelerated by an electric fan with wind speed of 3.5 m/s. In order to obtain a visible corrosion process, different circulation systems have been selected to obtain an appropriate rate of water penetration: for first eight cycles, specimen was wetted for 6 h and dried for 18 h. Then, the next 8–29 cycles specimen was wetted for 6 h and dried for 42 h. For last 30 cycles, specimen is wetted for 12 hours and dried for 60 h.

### 2.5. Ag/AgCl Electrode Theoretical Background

The Ag/AgCl chloride electrode is one solid-state membrane electrode which usually consists of a silver core with a coating of AgCl. Due to such coating has a low solubility, AgCl tends to be saturated in the electrolyte. When the overall ionic strength of the aqueous solutions is low, chloride ion activity and concentration are approximately equal. The relationship of the interfacial potential of AgCl electrode and chloride ion activity can be expressed by Nernst’s law [10,37], as Equation (1). The chloride concentration can be obtained by Equation (2).

(1) $$E_{\mathrm{Ag}/\mathrm{AgCl}}=E_{\mathrm{Ag}/\mathrm{AgCl}}^{0}-\frac{\mathrm{RT}}{\mathrm{nF}}\mathrm{In}[\alpha_{\mathrm{Cl}-}]$$

(2) $$\alpha_{\mathrm{Cl}-}=e^{\frac{\mathrm{nF}}{\mathrm{RT}}(E_{\mathrm{Ag}/\mathrm{AgCl}}^{0}-E_{\mathrm{Ag}/\mathrm{AgCl}})}$$

where, $E_{\mathrm{Ag}/\mathrm{AgCl}}$ represents the actual measured value, which is the measured open-circuit potential of AgCl electrode versus a reference electrode. $E_{\mathrm{Ag}/\mathrm{AgCl}}$ represents standard potential of Ag-AgCl equilibrium. R is the gas constant, which is 8.3144 J/(K·mol). T is the absolute temperature (K). F is the Faraday Constant, which is 96.487 KJ/(V·mol). n is the valence. $\alpha_{\mathrm{Cl}-}$ is the activity of the chloride ions in solution. Actually, the calibration curves can be simplified by linear regression analysis according to following Equation (3):

(3) $$E_{\mathrm{Ag}/\mathrm{AgCl}}=m\;\mathrm{In}[\alpha_{\mathrm{Cl}-}]+b$$

A high concentration of hydroxide ion, such as pore solution of cement-based materials, may induce interference in the potentiometric determination of the chloride concentration with an Ag/AgCl electrode [27]. The chloride concentration can be calculated by employing the activity coefficient γ_Cl−_ [28], which can been expressed by Equation (4).

(4) $$\alpha_{\mathrm{Cl}-}=C_{\mathrm{Cl}-}\cdot \gamma_{\mathrm{Cl}-}$$

Many studies have been done to get γ_Cl−_ [28,40]. According to literature [40], the γ_Cl−_ was calculated for expressed pore solution and when the chloride concentration from 0.1 to 1.0 M γ_Cl−_ value is from 0.656 to 0.604.

## 3. Results and Discussion

### 3.1. Calibration in Solution

**Figure 3:**
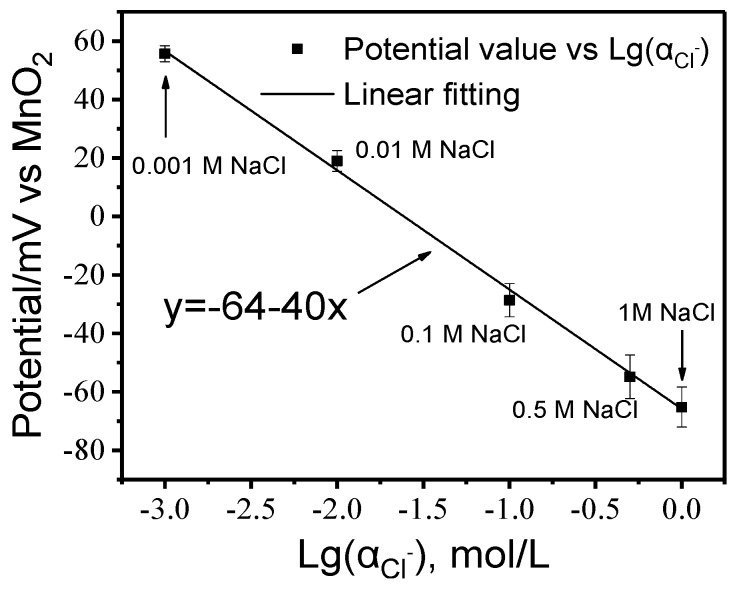
The calibration curve of Ag/AgCl in synthetic pore solution.

The potentiometric response behaviors of Ag/AgCl electrodes with increasing chloride concentration at 20 ± 2 °C are exhibited in Figure 3. It is apparent that there is an excellent agreement between potentials of Ag/AgCl electrodes and their corresponding linear fitting, with a correlation coefficient of 0.99. This is a clear indication that the prepared Ag/AgCl electrodes have well working performance. The calibration curves can be expressed by the linear regression equation in Equation (2), which is also shown in Figure 3.

### 3.2. Application of Ag/AgCl Electrode in Mortar

**Figure 4:**
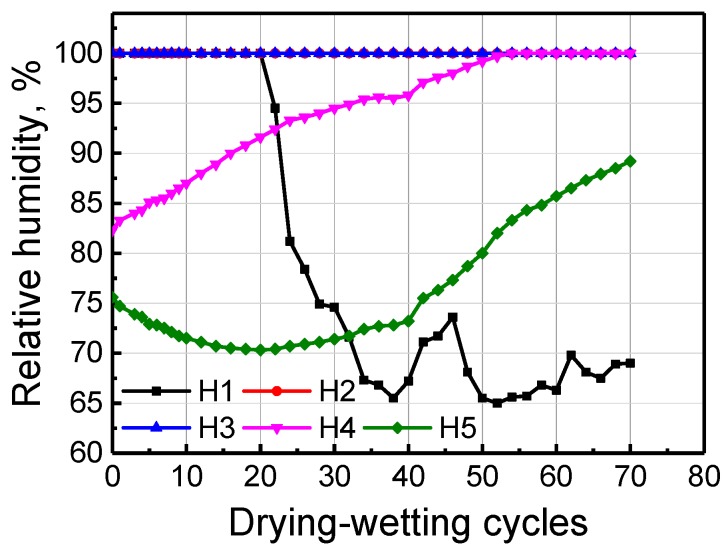
Development of interior relative humidity of mortar with dry-wet cycles.

**Figure 5:**
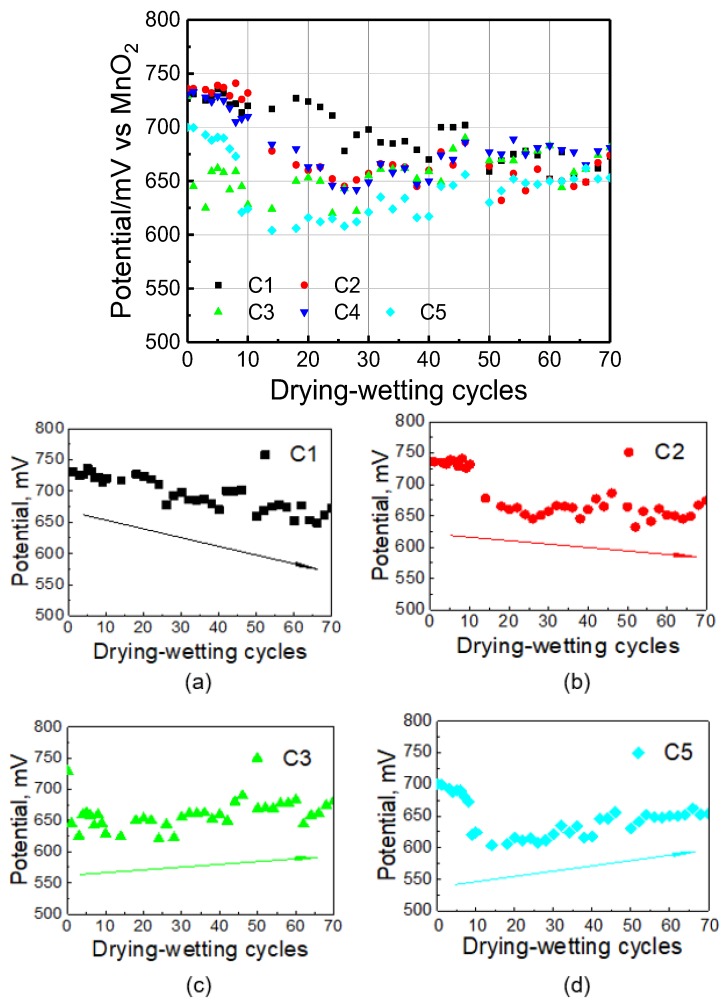
Potential evolution with the development of dry-wet cycles of Ag/AgCl electrodes in mortar sample.

The chloride ions are introduced into the mortar sample using dry-wet cycle by 5% NaCl solution. Because water is an effective carrier of chloride ions, the chloride penetration depth can to some extent be reflected by the humidity sensor. The humidity conditions of mortar with the same depth as Ag/AgCl electrodes are depicted in Figure 4. It illustrates that water can easily reach H1–H3 humidity sensor, but it is of relative difficulty to reach H4 and H5. Potential evolution of Ag/AgCl electrode to chloride ion in mortar with dry-wet cycle development is depicted in Figure 5. Although the potential responses of Ag/AgCl electrodes to Cl^−^ in synthetic pore solution with various chloride ion concentrations show good linearity, the obtained potential curves in mortar fluctuate remarkedly. The maximum fluctuation range between two adjacent potential values reached Ca. 100 mV. In fact, the potential curves obtained by Ag/AgCl electrode testing the continuous capillary absorption of chloride solution into concrete are relatively flat; therefore, this indicates that the dry-wet cycle does influence the testing error of Ag/AgCl electrode.

Furthermore, it is observed that as dry-wet cycle increases from 10 to 70, potential values exhibit evident overall upward or downward trend. Downward of potential values means increase of chloride concentration and lowering of slope the faster the chloride concentration increases; conversely, the concentration of chloride ions will decrease. The result of Figure 5 illustrates that chloride concentration at installation depth of C1 and C2 increases continuously. With regard to C3–C5, the slope of curves tends to increase, and this can be explained by a decrease in the free chloride content caused by physical adsorption and chemical binding of chloride ions by hydration products. In addition, because the sample was only cured with sufficient water for 15 days, there existed many unhydrated cement particles. Continuous hydration may lead to the increase of concrete alkalinity. Even for fresh concrete, the pH value is able to reach to 12.5 [7]. High concentration hydroxide has a certain inhibitory effect on activity of chloride ions, and there is a certain competitive relationship between phase hydroxide and chloride ion [29]. When the concentration of chloride ions is relatively low in highly alkalinity solution, oxidation film will be formed instead of AgCl, leading to a decrease of potential. It is also noticed that during early cyclic period before 15 cycles, the curve fluctuates greatly. This is because AgOH layer might be formed due to low chloride concentration [8]. Silver hydroxide is unstable and tends to convert to argentous oxide and water as expressed in Equation (5) [41]:

(5) $$\mathrm{AgOH}⇄\mathrm{AgO}+H_{2}O$$

Moreover, it is obvious that the potential is relatively stable after 15 cycles, which is due to the sensor being able to recover quickly once it comes into contact with chloride ions [28].

**Figure 6:**
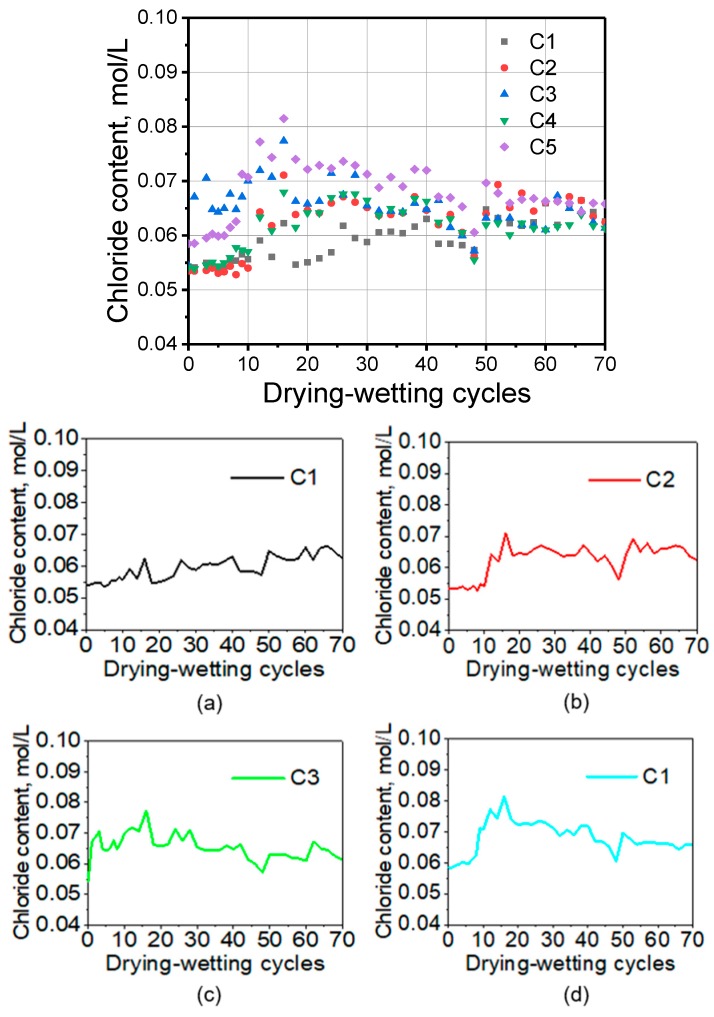
Chloride concentration calculated by Nernst’s law with the absence of temperature correction.

Regarding the results in Figure 5, the chloride concentration can be calculated by the linear function in Figure 3. The activity coefficient γ_Cl−_ was approximately selected as 0.6 according to literature [40]. It can be observed that the chloride concentration displays an increasing trend at the early age but shows a different trend after 15 cycles as shown in Figure 6. The chloride concentration at C1 depth continued to increase, while that at C2 depth was relatively stable. However, the concentration of chloride ions at relatively deep depths showed a trend of continuous decline. The continuous decrease in C5 is mainly attributed to the fact that chloride ions cannot enter the mortar sample in large quantity in a short time. Moreover, the original ions gradually decrease due to the physisorption and chemisorption. The main product of chemisorption is the so-called Friedel’s salt [32]. In general, the change of chloride concentration should be sensed much earlier by the outer-layer AgCl electrode, but there is no obvious increase trend of C1. This phenomenon can be attributed to the heterogeneity of cement mortar or the cracks. In this case, the AgCl electrode with a deeper depth responds slowly. This also indicates the limitation of AgCl electrode that it can only detect the concentration of chloride ions in a small domain.

**Figure 7:**
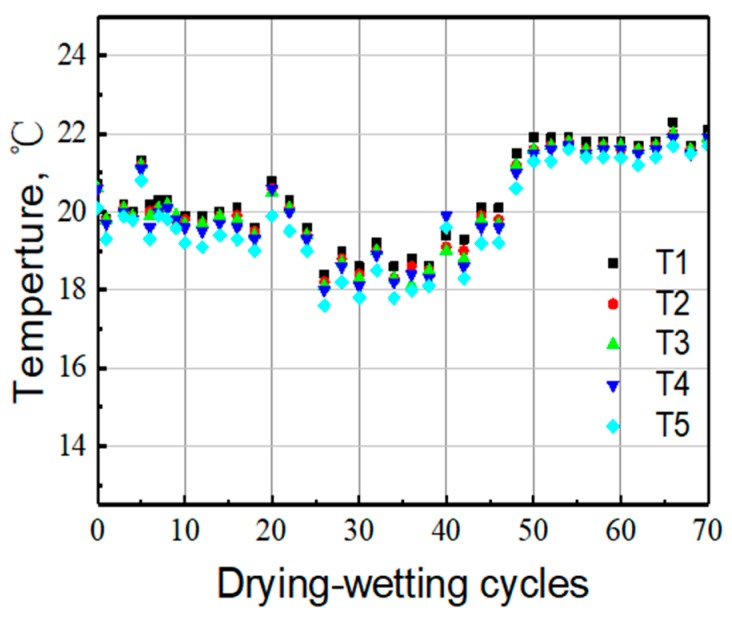
Temperature evolution with the development of dry-wet cycles by the humidity sensor in mortar sample.

**Figure 8:**
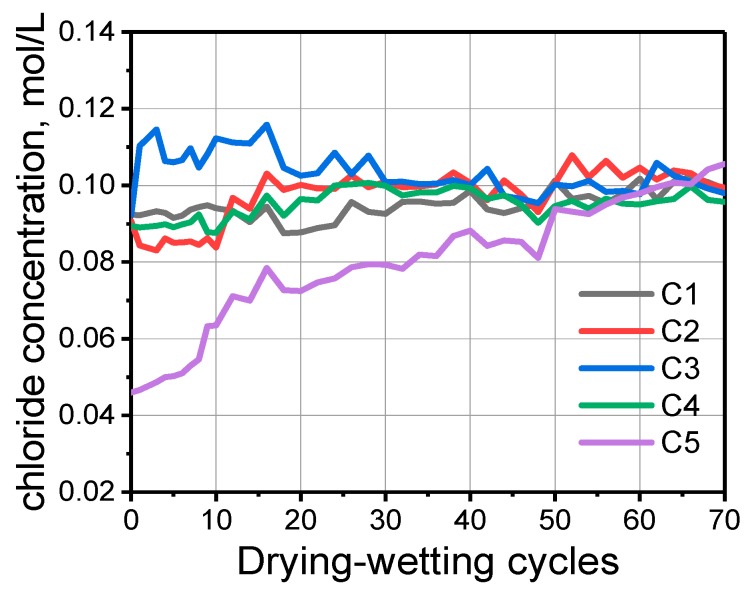
Chloride concentration calculated by Nernst’s law with presence of temperature correction.

**Figure 9:**
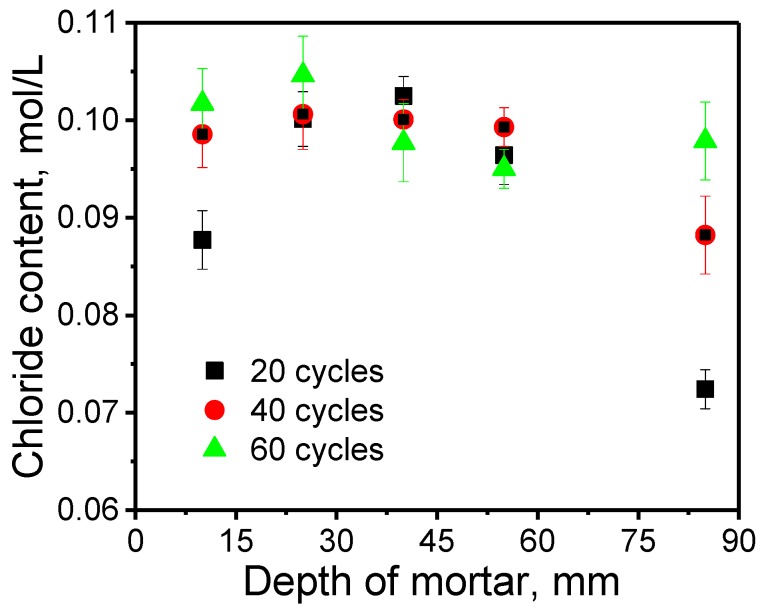
The chloride distribution at 20, 40, 60 cycles.

In Figure 7, the temperature of the tested position of each Ag/AgCl electrode is obtained by the temperature sensor. The influence of temperature on the Ag/AgCl detected result is remarkable according to the Nernst’s law. In order to investigate the influence of temperature on potential value, the chloride concentrations in the presence of temperature correction were calculated. The results are shown in Figure 8. After the temperature correction, the data dispersion is relatively smaller. However, the chloride concentration values according to C1–C4 are high at the beginning of the water penetration process. This is because the cracks in the sample provide the water channel as shown in Figure 4. Extracting the data at 20, 40, 60 cycles, Figure 9 can be obtained. It illustrates the distribution of chloride ions related to the depth. It can be seen that the concentration of chloride is obviously lower at deeper depths. However, at the same depth, the concentration value in different cycles varies little, due to the fast water penetration process at early stages.

### 3.3. Error Source in Potentiometry of Solution and Mortar

The chloride ion sensor has high sensitivity and selectivity to chloride ion concentration. However, results are influenced and restricted by many factors, e.g., interfering ion, high pH, temperature. In addition, the preparation method of Ag/AgCl electrode and installation distance from the reference electrode will affect the final test results [19].

Considering above interfering factors, the potential response curve is linear in solution, but fluctuates markedly in mortar. In previous literature [28], the performance of the electrode is calibrated by adding NaCl solution into fresh mortar. Such curves are relatively flat as well. In this study, chloride ions are introduced into cement mortar through dry and wet circulation, which is more similar to actual engineering conditions. The results illustrate that dry-wet cycles have a great influence on the accuracy of electrode test results, but the variation rule of chloride ion can still be obtained through a large amount of data. The reason for this effect may be caused by the continuous variation of the internal stress and strain environment of mortar caused by the alternations of wet and dry or the change of ion concentration in the pore solution, which needs to be further studied.

It can be seen that the potential responses Ag/AgCl electrodes are influenced by hydration throughout total service life in cement sample. It is characterized by violent potential fluctuations in the early hydration period and a continuous potential decline in dependence on adsorption of hydration products such as calcium silicate hydrate. It seems that the corresponding datum is meaningless in the early hydration period. Moreover, if accurate value is required, the effect of hydration and hydration products needs to be considered. Potential response of Ag/AgCl electrodes for both absence or presence of Cl^−^ interference in cement sample irregularly oscillates during total test procedure. This manifests that Ag/AgCl sensors are of little value for quantitatively determining the free chloride concentration in mortar pore solution during dry-wet cycles, and it seems only able to indicate qualitative variation trends of chloride.

### 3.4. A Comparison of ALS and Ag/AgCl Electrode in Mortar

ALS used as corrosion sensor has been widely applied in various reinforced concrete structures. In marine environment, chloride is the main reason initiating corrosion potential change of anodes since once the chloride concentration in the spatial domain around the anodes reaches a threshold value, the anode will turn into active state, resulting in a significant change of voltage and current between the anode and cathode. When the metal holders are mounted to the embedded steel, six anodes, which are made of common carbon steel, have different distance from the sample surface. According to the depassivation signals, one by one during the penetration of the aggressive substance, the depassivation front can be traced, and the process of chloride penetration can be probably predicted. A simple comparison of ALS and Ag/AgCl electrode in mortar is conducted to give some comprehensive understanding of performance of Ag/AgCl electrode under dry-wet cycle condition.

**Figure 10:**
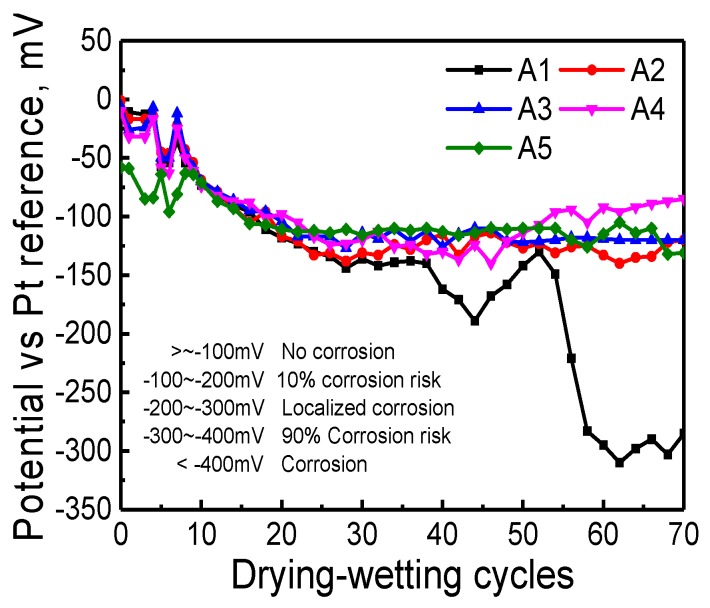
Potential (against Pt reference) evolution with the development of dry-wet cycles of ALS in mortar sample.

**Figure 11:**
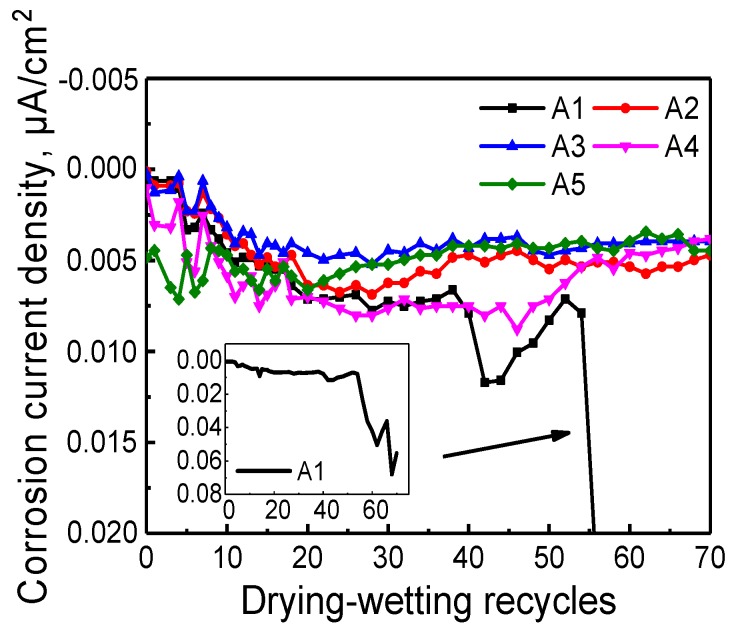
Current evolution with the development of dry-wet cycles of ALS in mortar sample.

Potential evolution of each anode with the development of dry-wet cycles in mortar sample can be observed from Figure 10. That all potential values are totally negative illustrates that the anode potential is less than the cathode potential and anodic steel reinforcement prone to lose electrons, leading to the depassivation of the steel surface. The corrosion risk can be assessed by corrosion potential according to corrosion standard for reinforcement in China, which is also dispalyed in Figure 10. It can be deduced that except A1, the corrosion risk of all steel is less than 10%. A1 initiates corrosion at the 51 cycles and reaches potential equilibrium at 60 cycles. Current evolution with the development of dry-wet cycle of ALS in mortar sample is shown in Figure 11 and in such a manner that the corrosion current is defined as the positive current. The negative current represents the corrosion current of the reinforcement, indicating that the reinforcement embedded in the mortar is prone to corrosion. The variation tendency of current accords well with potential curve. At the initial phase, curve oscillates due to mortar interior environmental changes caused by cement hydration. This negative current indicates that the electron is moving towards the cathode, and the anode is prone to be corroded. From Figure 11, it can be seen that there is a significant increase of the negative corrosion current density of A1 after 50 cycles.

Although ALS has been widely used in various projects, it has many unconquerable limits. For example, the results from ALS just displayed the corrosion behavior of adopted material, not the steel used in cement-based material, resulting in a certain difference. In some cases requiring accurate prediction or monitoring, this error is unneglectable.

It can be seen that both ALS and Ag/AgCl electrode have an unstable period in the early stage. Then the curves of both the potential and the current became relatively flat until the cycles reach about 50 cycles. After around 50 cycles, the corrosion potential and the current of A1 increase significantly. The sudden shift of potential indicated that the chloride concentration on the surface of steel has reached a critical value leading to depassivation of the passivation film. Because the corrosion signal of ALS is the corrosion potential of the steel bar depassivation, the concentration of chloride ion has reached the critical value of depassivation, which illustrates that ALS has a certain hysteresis about chloride concentration change when compared to Ag/AgCl electrode. The potential of Ag/AgCl electrode is affected by the chloride ions in the solution. In comparison, for the ALS, the potential will only change if the concentration of chloride ions around it reaches a certain value, while the corrosion potential can only be obtained by anode ladder. In theory, the critical chloride content can be obtained by the combination of ALS and silver chloride electrode. However, it seems that the obtained critical chloride content has a certain error because of the interference from dry-wet cycle.

## 4. Conclusions

In the present work, Ag/AgCl electrodes were prepared using continuous current and were calibrated in synthetic pore solution. The performance of Ag/AgCl electrodes in reinforcement corrosion monitoring is evaluated in mortar. The main conclusions are summarized as follows:

The dry-wet cycle greatly promotes the permeability and transmission of water and chloride ions to the cement mortar. During the process of chloride, ions penetrate into the cement-based materials; in situ monitoring by mean of Ag/AgCl electrode to some extent can reflect chloride content under dry-wet cycle condition. However, the dispersion of testing results is significantly affected by the dry-wet cycles. In addition, temperature correction is necessary for obtaining a more precise potential value. Finally, in terms of detecting the chloride ions, the Ag/AgCl is more sensitive than the ALS.

## Figures and Tables

**Table 1:** Embedded depth of sensors.

Sensor	Depth
H1/C1/T1/A1	10 mm
H2/C2/T2/A2	25 mm
H3/C3/T3/A3	40 mm
H4/C4/T4/A4	55 mm
H5/C5/T5/A5	85 mm
ALS cathode (carbon steel)	100 mm
ALS reference electrode of (Pt.)	55 mm

Notes: H is the humidity sensor; C is the AgCl/Ag electrode; T is the temperature sensor; A is the anode ladder monitoring system.
